# Sugarcane under siege: integrated physiological, molecular responses and mitigation strategies to salinity and drought stress

**DOI:** 10.3389/fpls.2026.1915940

**Published:** 2026-08-10

**Authors:** Krishan K. Verma, Xiu-Peng Song, Qiang Liang, Lin Xu, Hai-Rong Huang, Dong-Liang Huang, Yang-Rui Li

**Affiliations:** Guangxi Key Laboratory of Sugarcane Genetic Improvement, Key Laboratory of Sugarcane Biotechnology and Genetic Improvement (Guangxi) of Ministry of Agriculture and Rural Affairs, Sugarcane Research Institute, Guangxi Academy of Agricultural Science, Nanning, Guangxi, China

**Keywords:** CRISPR/Cas9, photosynthetic leaf gas exchange, phytohormones, reactive oxygen species, *Saccharum* spp., stress adaptation

## Abstract

Saline soil and drought are among the most devastating abiotic stresses constraining sugarcane (*Saccharum* spp.) production globally, with soil salinity affecting over 1,125 Mha worldwide and drought causing severe yield losses in tropical and subtropical agroecosystems. As a glycophytic C_4_ crop supplying ~80% of the world’s sugar, sugarcane is particularly vulnerable, with threshold salinity tolerance at a mere 1.7 dS m^–1^ electrical conductivity (EC). This review integrates recent developments in the physiological, biochemical, and molecular responses of sugarcane during stress conditions. Under salinity, photosynthetic CO_2_ efficiency, chlorophyll integrity, source–sink partitioning, reactive oxygen species (ROS) metabolism, phytohormone signaling, and osmolyte accumulation are altered based on the sugarcane cultivars and cultivation regions. Under drought, stomatal regulation, root hydraulics, abscisic acid (ABA) cascades, and the expression of dehydrin and late embryogenesis abundant (LEA) proteins govern tolerance. At the molecular level, ion-transporter genes (*SOS* pathway), *DREB/ERF* transcription factors, aquaporins, and small RNAs constitute central regulatory hubs. Mitigation strategies, including agronomic interventions, exogenous osmoprotectants, plant growth-promoting rhizobacteria, biochar amendment, and advanced breeding tools such as CRISPR/Cas9, marker-assisted selection, and transgenic approaches, are comprehensively discussed for sustainable sugarcane production.

## 1 Introduction

### 1.1 Global importance of sugarcane and abiotic stress challenge

Sugarcane is a major agro-industrial crop worldwide. It accounts for about 80% of global sugar production, which significantly supports rural economies and provides large-scale employment for rural socio-economic development (Mall et al., 2025). Sugarcane has emerged as a byproduct, such as ethanol, waxes, fibers, biofertilizers, animal feed, paper, plywood, and bioenergy (Iqbal and Saleem, 2015). Sugarcane has the capacity for optimal conversion of light to sucrose and optimal nitrogen and water-use efficiency (Byrt et al., 2011). The production and productivity of sugarcane have increased in recent years; demand for higher cane yields has also risen, along with demand for raw materials for other agroindustries. Furthermore, sugarcane biomass serves as a critical component in the emerging circular bioeconomy paradigm, where its cellulosic byproducts are transformed into sustainable energy sources, biochemical compounds, and value-added materials, consequently minimizing environmental waste and greenhouse gas emissions (Wang et al., 2025). Environmental stresses are major threats to sugarcane crop development globally (Mall et al., 2025). The interactive effects of different abiotic stresses severely influence crop growth, development, and production, thereby affecting the photosynthetic apparatus system II (PS II), biochemical disruptions, and molecular alterations in sugarcane (Kumari et al., 2023; Singh et al., 2023).

Salinity and drought are the severe threats to sugarcane productivity, and their co-occurrence is increasingly common as a consequence of climate change, groundwater depletion, and the expansion of irrigation-based agriculture into marginal lands. Soil salinization, the excessive accumulation of salt in soil, degrades crop production, ecosystem function, and soil fertility, threatening sustainable agriculture and food security. Food production significantly influences land use and the use of natural resources. The estimated area of salt-affected soils was about 1381 Mha, representing ca. 10.7% of the total global land area, which has a significant impact on agriculture worldwide (FAO, 2024; United Nations Office for Disaster Risk Reduction (UNDRR) & International Science Council (ISC), 2025).

Sugarcane requires approximately 1500–2500 mm of water per growing season, with the grand growth phase requiring ~550 mm; any deficit during this phase disproportionately suppresses internode elongation and sucrose accumulation. Drought and salinity frequently co-occur; salt-affected soils are often found in dryland or poorly irrigated environments, and stresses share similar physiological consequences, particularly osmotic stress and oxidative damage, though their specific molecular responses differ significantly. Sugarcane has evolved various adaptive strategies, i.e., escape strategy (ineffective given its long growing season), avoidance (stomatal closure, leaf rolling, reduced leaf area), and tolerance (the maintenance of cellular homeostasis through ion compartmentalization, antioxidant upregulation, osmolyte synthesis, and stress-responsive gene expression) (Gujjar and Supaibulwatana, 2019). Tolerant genotypes employ a sophisticated ensemble of molecular regulators, including the Salt Overly Sensitive (SOS) pathway, DREB/ERF transcription factors, aquaporins, and small RNA networks, to reprogram metabolism under stress conditions (Theerawitaya et al., 2020; Wang et al., 2026a).

This review synthesizes recent developments on sugarcane responses to salinity and drought stress, covering agronomic characteristics, physiological traits, and molecular responses. The mitigation strategies, ranging from agronomic management and exogenous chemical application to conventional breeding and cutting-edge biotechnology, are critically assessed. It concludes by identifying research gaps and outlining directions for developing stress-resilient sugarcane cultivars suitable for challenging agro-climatic conditions.

## 2 Physiological responses of sugarcane to salinity and drought stress

Soil salinity and drought impose two intertwined primary injuries on sugarcane, such as osmotic stress and ionic toxicity (salt-specific), arising from the accumulation of Na^+^ and Cl^–^ ions in photosynthetic tissues. Together, these perturbations cascade into a suite of physiological dysfunctions that span from the subcellular (disruption of electron transport chains) to the whole-plant level (suppressed biomass accumulation and sucrose yield). **Table 1** summarizes key physiological parameters and their responses to varying salinity and drought stress severity.

**Table 1:** Summary of key physiological responses in sugarcane plants under salinity and drought stress conditions.

Physiological characteristics	Stress condition	Impact	Source
Photosynthetic CO_2_ assimilation rate	Salinity	As a consequence of restricted internal CO_2_ availability and non-stomatal metabolic limitations, the photosynthetic CO_2_ assimilation rate declines severely by 16.85 to 91.44% across diverse genotypes.	Chiconato et al., 2019; Cruz et al., 2018; Dhansu et al., 2022
	Drought	The downregulation was significantly lower in YZ05-51 (38.80%) than in YT93-159 (52.81%) and in GT42 sugarcane cultivars.	Ai et al., 2025; Verma et al., 2021a, Verma et al., 2021b
Stomatal and transpiration traits	Salinity	Under salinity levels ranging from EC 4 to 12 dS m^−1^, stomatal conductance (gs) drops by 14.96 to 84.25%. This stomatal closure directly reduces transpiration rate (E) by 14.13-89.8%, limiting the plant’s capacity for nutrient transport and evaporative cooling.	Dhansu et al., 2022; Sharma et al., 2021; Cruz et al., 2018
Maximum quantum yield of PSII (Fv/Fm)	Salinity	The maximum quantum efficiency of Photosystem II (Fv/Fm) decreases by 5.33 to 42.67% under the EC of 4–12 dS m^−1^. This reduction signals severe photoinhibition and structural damage to the thylakoid membranes.	Chiconato et al., 2019; Vu et al., 2023
	Drought	Significantly reduced under a limited water supply, indicating damage to the photosynthetic apparatus.	Ai et al., 2025
Photosynthetic pigments	Salinity	Prolonged saline irrigation degrades total chlorophyll content (SPAD values drop by 8.46 to 56.7%). Under combined salinity and drought conditions, total photosynthetic pigments and carotenoids decrease in all genotypes, reducing overall light-harvesting capacity (LHC).	Dhansu et al., 2022; Apon et al., 2023. Mahadevaiah et al., 2023; Tang et al., 2021
	Drought	Significantly downregulated in different sugarcane genotypes under stressed conditions.	Zhang et al., 2020
Relative water content (RWC)	Drought	Progressive water deficits cause a sharp decline in leaf RWC, with the magnitude of the drop directly proportional to the soil water potential deficit and highly dependent on cultivar tolerance.	Gujjar and Supaibulwatana, 2019
Sucrose phosphate synthase activity	Salinity	At the biochemical level, NaCl stress inhibits Sucrose Phosphate Synthase (SPS) activity in source leaves. This down-regulation impairs sucrose synthesis and export, failing to satisfy the sink demand of developing culms.	Gomathi and Thandapani, 2004, Gomathi and Thandapani, 2005
Plant growth, development, production, and sugar content	Salinity	Salinity levels exceeding an EC of 4 dS m^−1^ significantly inhibit stalk height and stem elongation, primarily due to reduced turgor-driven cell expansion in the culm internodes. Sugarcane maintains a strict salinity threshold of EC 1.7 dS m^−1^. Every unit (dSm) increase above this critical baseline inflicts a 5.9% reduction in cane yield, accumulating up to an overall 50% loss in total sugar content under high-stress conditions.	Rao et al., 2015; Brindha et al., 2019; Watanabe et al., 2020
	Drought	Reduction was significantly lower in the drought-tolerant genotype YZ05-51 (40.39%) than in the sensitive genotype YT93-159 (52.15%), and stress-tolerant genotypes maintained higher dry biomass under stress.	Zhang et al., 2020; Ai et al., 2025

### 2.1 Photosynthetic efficiency and stomatal regulation

Photosynthetic response is the fundamental physiological process that is mainly caused by salinity and drought stress (Hussain et al., 2003). The adverse effects of salinity on photosynthetic CO_2_ assimilation rate are the primary reason for reduced culm elongation and juice quality of sugarcane (Watanabe et al., 2020). Multiple studies indicated significant reductions in photosynthesis (16.85 to 91.44%), stomatal conductance (14.96 to 84.25%), transpiration rate (14.13 to 89.8%), and chlorophyll fluorescence efficiency (5.33 to 42.67%) during stress conditions. The net photosynthetic rate correlates strongly with stomatal conductance, intercellular CO_2_ concentration (C_i_), and the activity of photo-assimilate enzymes. Under salinity, sugarcane initiates a rapid stomatal closure response mediated by ABA signaling and guard-cell ion dynamics to limit water loss, but this simultaneously restricts CO_2_ diffusion to the carboxylation site of phosphoenolpyruvate carboxylase (PEPCase) and ribulose-1,5-bisphosphate carboxylase/oxygenase (RuBisCO), suppressing carbon fixation (Sharma et al., 2021; Cruz et al., 2018; da Silva et al., 2022).

**Figure 1:**
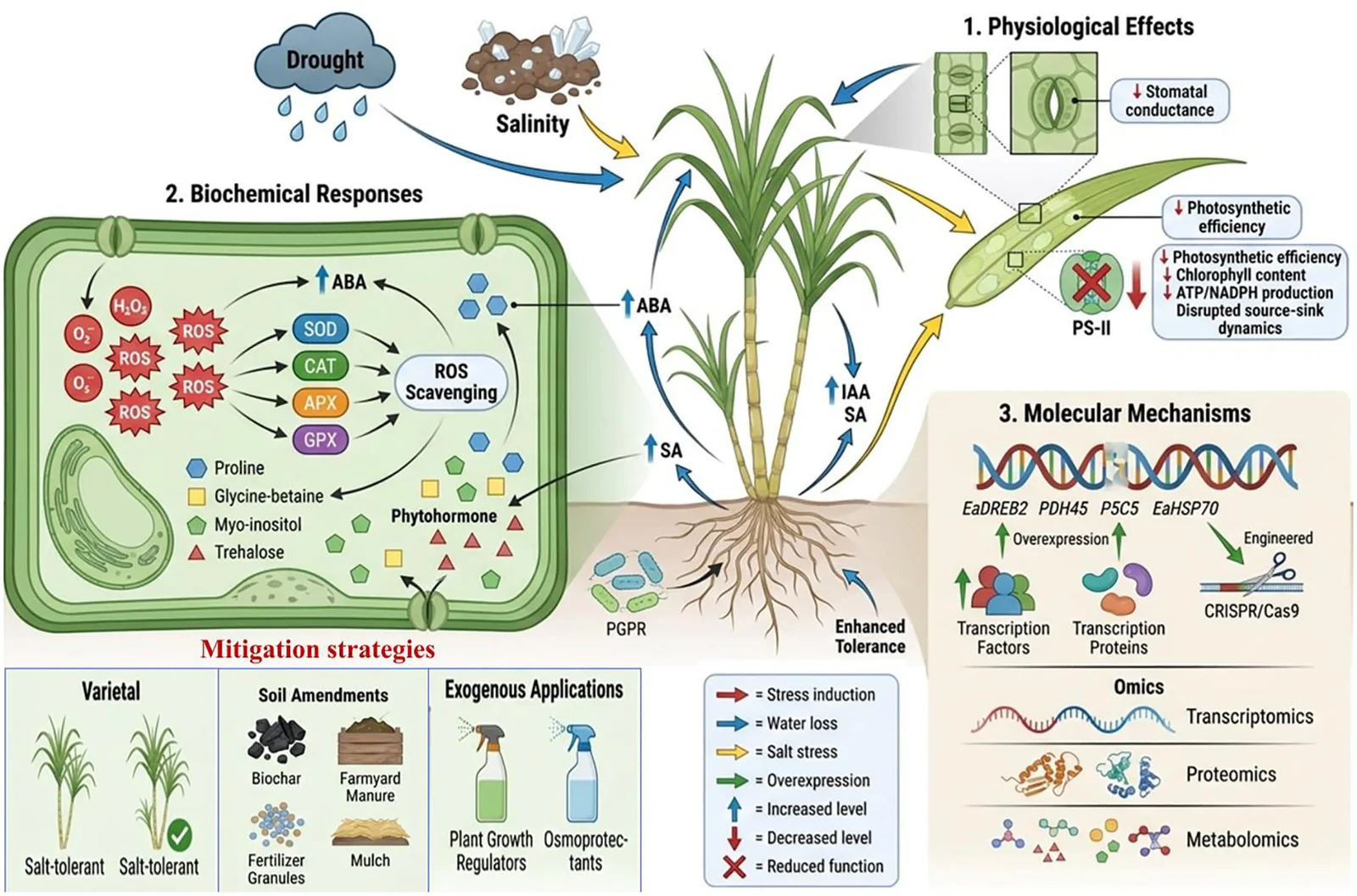
Schematic overview of the physio-biochemical and molecular responses of sugarcane (*Saccharum spp.*) to drought and salinity stress, and associated mitigation strategies. Environmental stress induction pathways showing the impact of drought (blue arrows; water loss) and salinity (yellow arrows; ionic/osmotic stress). (1) Physiological consequences include reduced stomatal conductance, impaired Photosystem II (PS-II) function, decreased chlorophyll content, and disrupted source-sink dynamics; (2) biochemical responses encompassing ROS (O₂⁻, H₂O₂) overproduction, antioxidant enzyme activation (SOD, CAT, APX, GPX), osmoprotectant accumulation (proline, glycine betaine, myo-inositol, trehalose), and phytohormone (ABA, IAA, SA) signaling; and (3) molecular responses include overexpression of stress-responsive genes (*EaDREB2, PDH45, P5CS, EaHSP70*), CRISPR/Cas9-mediated genome editing, and integrated omics approaches (transcriptomics, proteomics, metabolomics). Applied mitigation strategies, i.e., selecting stress-tolerant varieties/cultivars; soil amendments (biochar, farmyard manure, fertilizers, and mulch); and exogenous application of plant growth regulators and osmoprotectants.

Under drought, stomatal closure is the first physiological response, driven by the accumulation of ABA in root and guard cells as leaf water potential declines. The activation of the *SoNCED* (9-cis-epoxycarotenoid dioxygenase) gene in sugarcane upregulates ABA biosynthesis, triggering K^+^ and Ca^2+^ efflux from guard cells and consequent stomatal aperture reduction (Gujjar et al., 2020). While this limits water loss, the concomitant reduction in C_i_ and the over-reduction of photosynthetic electron carriers progressively impair the light- and dark-phase reactions of photosynthesis (Simões et al., 2019; Verma et al., 2022) (**Figure 1**).

The photosystem II (PSII) complex is particularly sensitive to both stresses. Salinity reduces the maximum quantum yield of PSII (Fv/Fm) in sugarcane, reflecting damage to the reaction center D1 protein and the oxygen-evolving complex by up to ~43% at high salinity (Chiconato et al., 2019; Vu et al., 2023). Reduced Fv/Fm compromises the electrochemical potential gradient for ATP synthase, decreases ATP and NADPH generation, and ultimately depresses the Calvin cycle (Simões et al., 2019).

### 2.2 Chlorophyll content and photosystem-II integrity

Chlorophyll serves dual functions, such as harvesting photosynthetically active radiation and maintaining the structural integrity of the thylakoid membrane. Under salinity, chlorophyll concentration declines through various mechanisms, such as direct ionic toxicity of Na^+^ and Cl^–^ on the chloroplast ultrastructure, enhanced chlorophyllase activity coupled with suppressed 5-aminolevulinic acid (ALA) synthase in sensitive genotypes, and ROS-mediated peroxidation of the pigment-protein complexes (Tang et al., 2021; Dhansu et al., 2022). SPAD values in salt-stressed sugarcane dropped by 56.7% relative to control plants, with the magnitude of decline correlating with stress intensity and genotype sensitivity (Sharma et al., 2021; Apon et al., 2023) (**Figure 1**).

Under drought, the mechanisms of chlorophyll loss partially overlap with those under salinity, mainly through ABA-induced stomatal closure, reduced C_i_, and ROS generation. However, the initial trigger is osmotic dehydration rather than ion accumulation. Vu et al. (2023) demonstrated temporal responses in sugarcane under progressive salinity stress. They found that chlorophyll concentration declined during salt treatment. In contrast, Fv/Fm depression appeared only from week 8 onward, indicating that the photochemical apparatus is initially more resilient than chlorophyll biosynthetic machinery (Deng et al., 2023).

### 2.3 Water relations, osmotic adjustment, and root hydraulics

Salt stress reduces the osmotic potential of the soil solution, creating a water-deficit condition analogous to physiological drought even when soil moisture is adequate. The consequent decline in leaf relative water content (RWC) triggers a cascade of cellular dehydration events, including loss of turgor, plasmolysis of sensitive cells, and impairment of enzyme-catalyzed reactions. Excess soil salinity also interferes with water movement through root cells by reducing aquaporin activity and altering the hydraulic conductivity of the root apoplast (Simões et al., 2019; Chiconato et al., 2021). Under drought stress, the reduction in soil water potential is the primary driver of reduced RWC, and sugarcane responds by closing stomata (mediated by ABA), accumulating cytoplasmic osmolytes (proline, glycine betaine, trehalose), and modifying root architecture to enhance water extraction. Root-to-shoot ABA signaling is the dominant long-distance chemical signal under both stresses, integrating soil water status with above-ground stomatal responses (Gujjar and Supaibulwatana, 2019).

Osmotic adjustment, the active reduction of cell osmotic potential through solute accumulation, allows plants to maintain turgor and continue metabolic activities at low water potential. In sugarcane, the accumulation of proline and sucrose, inorganic ions (in vacuoles), and other compatible solutes in the cytosol constitutes the primary osmotic adjustment mechanism. Tolerant genotypes maintain higher osmotic adjustment capacity than sensitive ones, enabling continued expansion growth even under moderate salinity or drought stress (**Figure 1**).

## 3 Role of antioxidant activities on sugarcane plants during stressed conditions

When sugarcane is subjected to drought, high temperatures, or salinity, it produces reactive oxygen species (ROS) as a natural byproduct of its metabolism. While high levels cause oxidative damage, these molecules also act as vital signaling agents that trigger the plant’s defense mechanisms (Xie et al., 2019; Silva et al., 2025). This excessive increase in ROS disrupts the cells’ redox potential. Free radicals are highly toxic and can disrupt essential physiological and biochemical pathways involved in synthesizing compounds necessary for sugarcane growth and development, including nucleic acids, ATP, proteins, and hormones (Heyno et al., 2011; Moreira et al., 2020). The antioxidant defense system produced within sugarcane plant cells helps to tolerate salt stress by scavenging ROS. The accumulation of proline, sucrose, glycine betaine, and other solutes in the cytoplasm helps achieve osmotic adjustment, thereby increasing salt tolerance (Hasanuzzaman and Tanveer, 2020).

However, specific enzymes, i.e., POD, CAT, and SOD, are essential for ROS detoxification. It indicated that sugar plants have developed a complex and effective antioxidant system to mitigate the adverse effects of salt-induced oxidative stress. The relationship between ROS signaling and modulation of antioxidant enzymes also enhances salt tolerance through transgenic or marker-assisted breeding approaches (**Figure 1**).

Generally, free radical scavenging processes in plants are directly associated with enzymatic and non-enzymatic antioxidant activities. Heme-containing CAT, found in peroxisomes, converts H_2_O_2_ into H_2_O and O_2_, reducing oxidative stress, and is active for long periods during salinity and water-deficit stress. Peroxidase is essential for scavenging H_2_O_2_ from SOD-catalyzed O_2_ dimethylation. Ascorbate peroxidase, the primary enzyme in the Ascorbate-Glutathione pathway, detoxifies H_2_O_2_ using ascorbate as an electron source. Catalase removes excess ROS during stress, whereas APX and POX presumably modulate ROS for signaling. A variety of crop species showed a positive association between antioxidant capacity and stress tolerance capacity (Hossain and Dietz, 2016).

Sugarcane plants under salt stress have demonstrated the ability to mitigate oxidative damage by augmenting their antioxidant defense systems. The significant increase in the CAT enzyme activity in sugarcane plants in response to NaCl-induced stress from the early growth stage to tillering stage (Pagariya et al., 2012). Salt stress leads to a notable increase in SOD activity, while simultaneously balancing CAT and APX activities in sugarcane plants (Medeiros et al., 2014; Mohanan et al., 2021). GPX improved the conversion of H_2_O_2_ or organic hydroperoxides into water or related alcohols (Patade et al., 2014).

## 4 Regulation of phytohormones and osmolytes

Endogenous phytohormones play a crucial role in facilitating plant growth and mediating plant responses to stress by regulating molecular and cellular processes (Fahad et al., 2015; Wani et al., 2016). A wide range of plant hormones, including indole-3-acetic acid (IAA), gibberellins (GA), cytokinins, salicylic acid (SA), jasmonic acid (JA), nitric oxide, and abscisic acid (ABA), are affected by salt stress (Singh et al., 2023). Under salinity and drought stress, plant roots act as the primary sensors for sugarcane. They rapidly generate and accumulate elevated levels of ABA, a primary stress hormone that triggers defense mechanisms (Nong et al., 2024; Park et al., 2016). This results in stomatal closure and inhibits water loss by upregulating the *9-cis-epoxycarotenoid dioxygenase* (*soNCED*) gene in sugarcane (Gujjar et al., 2020). The efficacy of IAA is well acknowledged for stimulating plant development. However, as salt stress progressed, hormone levels decreased due to various factors (Nong et al., 2022). Salicylic acid is associated with the production and regulation of pathogen-mediated protein complexes, and plays a significant role in mitigating the detrimental effects of salt and drought stresses (Apon et al., 2023) (**Figure 1**).

Osmolytes are small, non-toxic, compatible organic solutes, such as proline, glycine betaine, myo-inositol, and trehalose, which accumulate in plant cells to mitigate abiotic stresses. In sugarcane, they enhance tolerance by driving osmotic adjustment to maintain cell turgor, scavenging damaging ROS, and protecting essential proteins and membrane structures (Patade et al., 2011; Dhansu et al., 2026). Proline is a highly effective compatible osmolyte that plants rapidly overproduce when subjected to abiotic stresses (Hayat et al., 2012). It prevents water loss by regulating the osmotic potential between the cytoplasm and vacuole, allowing the plant to maintain cell turgor and water uptake. Proline acts as an antioxidant, neutralizing excess ROS to prevent severe oxidative damage to DNA, lipids, and cellular structures. It stabilizes cellular structures, proteins, and lipid bilayers, preventing electrolyte leakage and damage to cell organelles under adverse environmental conditions. Sugarcane buds are highly sensitive to salinity and drought stress, which causes toxic ion buildup, nutrient imbalances, and oxidative damage. Pretreating sugarcane bud chips with osmolytes like glycinebetaine (GB) or proline protects the tissues. Glycinebetaine serves as an effective inducer of stress tolerance (de Morais Hervatin et al., 2024; Genetu and Getahum, 2024).

Myo-inositol is a major nutrient for the physiological development of sugarcane. In sugarcane, it drives critical functions like cell division, cell wall biosynthesis, hormone storage and transport, and abiotic stress management (Wang et al., 2026). Elevated myo-inositol acts as a compatible solute, stabilizing proteins and membranes, and protecting sugarcane cells against osmotic stress. Trehalose serves as a vital osmoprotectant and signaling molecule in plants, maintaining cellular integrity and membrane stability under abiotic stress. In sugarcane, the trehalose-6-phosphate synthase (ScTPS) enzyme serves as a key marker and confers enhanced salt stress resistance (Mansour and Hassan, 2026). It interacts with membrane lipids and proteins to prevent denaturation and preserve cellular structures during osmotic and oxidative stress. Its intermediate, Trehalose-6-phosphate (T6P), acts as a master regulator, coordinating carbohydrate metabolism with the plant’s growth and stress response pathways (Hu et al., 2020).

## 5 Molecular omics approaches for sugarcane improvement

Omics technologies are transforming sugarcane breeding by unraveling the molecular mechanisms underlying drought and salinity tolerance. These integrated biotechnological approaches, encompassing genomics, proteomics, transcriptomics, and metabolomics, decode the genetic and biochemical basis of stress-adaptive traits, such as osmotic adjustment and ion homeostasis. By mapping these molecular pathways, omics-driven strategies accelerate the development of climate-resilient cultivars with superior stress tolerance and sustained sucrose productivity (Wang et al., 2026a).

### 5.1 Transcriptomics

The transcriptome represents the complete set of RNA transcripts present in a cell. It enhances the precise identification of RNAs that influence a plant’s phenotypic traits under varying climatic conditions. Under abiotic stress, plants activate diverse molecular strategies to survive adverse environments, with transcription factors (TFs) playing a central role. Advanced transcriptomics tools have been applied to the study of TFs in sugarcane and other crops. Among them, NAC proteins, a family of TFs defined by the conserved *NAM, ATAF1/2*, and CUC domains, regulate plant growth and confer tolerance to abiotic stresses across a wide range of species (Budzinski et al., 2019; Srithawong et al., 2016).

Belesini et al. (2017) investigated the transcriptomic activity of two sugarcane varieties (SP81–3250 and RB855453) under drought stress using the Illumina HiScanSQ platform. Their analysis revealed the upregulation of several genes implicated in drought adaptation, including ascorbate peroxidase, E3 SUMO-protein ligase SIZ2, MYB transcription factors, key flavonoid biosynthesis enzymes, such as coenzyme A ligase, and aquaporins (Ali et al., 2019). Various receptor-like protein kinases (RLKs) and their induction upon drought onset in drought-sensitive varieties have been reported; these RLKs, along with basic helix-loop-helix (bHLH) transcription factors, 1-aminocyclopropane-1-carboxylic acid oxidase (ACC oxidase) involved in ethylene biosynthesis, and several uncharacterized genes, are major players in drought sensing (Belesini et al., 2017). Pereira-Santana et al. (2017) leveraged next-generation sequencing to characterize the transcriptomic response of the cultivar Mex 69–290 to osmotic stress. The researchers noted that the tolerant cultivars exhibited upregulated gene expression associated with transcriptional regulation, reduced oxidative stress, carbohydrate metabolism, flavonoid biosynthesis, and the production of various secondary metabolites. Furthermore, genes involved in abscisic acid (ABA) biosynthesis, osmotic regulation, and heat stress responses were also significantly enhanced (Li et al., 2023).

### 5.2 Proteomics

Proteomics analyzes the complete protein repertoire of cells, tissues, or organisms to elucidate their functional roles. Proteins regulate the plant’s stress response, and changes in plant proteomes are an important area of research. Different phases, including the cell cycle, differentiation, and development, as well as abiotic stressors, can result in diverse expression patterns of the same gene (Conde and Kirst, 2022). Sugiharto et al. (2002) isolated and identified the drought-inducible gene *SoDip22* from drought-stressed sugarcane using two-dimensional gel electrophoresis (2-DE). They demonstrated that *SoDip22* functions in drought-stress adaptation in bundle sheath cells via an ABA-mediated signaling pathway. An 18-kDa protein was extracted, purified, and identified in sugarcane leaves under drought stress using 2D gel electrophoresis (Jangpromma et al., 2007). Co-expression of the dehydration-responsive element-binding 2 gene (*EaDREB2*), sourced from *E. arundinaceus*, alongside the pea DNA helicase gene (*PDH45*), conferred enhanced drought and salinity tolerance in transgenic sugarcane (**Figure 1**). To understand the impact of drought on protein profiling, two sugarcane cultivars (RB 72910, stress-tolerant, and RB 943365, susceptible) were grown under drought-stressed conditions. The abundance of proteins involved in photosynthesis, signaling, and regulation showed differential regulation between two sugarcane genotypes: they were either up- or downregulated in RB 72910 but consistently downregulated in RB 943365 (Augustine, 2017; Kumar et al., 2023).

Khueychai et al. (2015) identified different types of proteins responsive to drought across different sugarcane cultivars, including K86-161 (resistant) and B34-164 (susceptible). Their findings demonstrated that gene expression of fructose-1,6-bisphosphate aldolase, O_2_-liberating enhancer proteins, and superoxide dismutase (SOD) increased to a greater extent in various parts of K86-161; conversely, these proteins were found in lower quantities in B34–164 under drought conditions. Furthermore, Salvato et al. (2019) quantified proteins from drought-stressed sugarcane stalk nuclei using filter-aided sample preparation (FASP) and LC–MS/MS. However, 37 TFs that were associated with different protein domains, e.g., *NAC, C2H2* (*Cys2-His2*), *bZIP* (basic leucine zipper), *C3H* (Cysteine3Histidine), LIM (*LIN-11, Isl-1*, and *MEC-3*), Myb-related (myeloblastosis viral oncogene homolog), HSF (heat shock factor), and auxin response factor (ARF), were characterized in response to drought conditions. Pacheco et al. (2013) analyzed the differentially expressed proteome in sugarcane roots in response to salinity stress across different cultivars. They concluded that most proteins in the resistant cultivar were involved in developmental processes, carbohydrate metabolism, the ROS pathway, protein protection, and membrane stability.

### 5.3 Ionomics and metabolomics

Ionomics, the high-throughput and comprehensive quantification of elemental composition in plant tissues, such as Na⁺, K⁺, Ca²⁺, and Mg²⁺, is essential for understanding and managing sugarcane under drought and salinity stress. Under salinity stress, ion homeostasis becomes disrupted across cells; however, plants can adapt to drought and salinity through osmotic adjustment. Various strategies have been proposed to counter salinity and drought stress in sugarcane plants. In sugarcane, salinity stress was shown to induce physicochemical alterations in buds (Rasheed et al., 2016). Salinity stress triggers an overproduction of hydrogen peroxide, leading to its accumulation, and elevates Cl^−^ and Na^+^ concentrations in sugarcane plant tissues, while simultaneously reducing K^+^ and Ca^2+^ levels. This disrupts critical ionic ratios, specifically the Ca²⁺: Na⁺ and K⁺: Na⁺ ratios, and stimulates the synthesis of protective osmolytes in sugarcane plant cells.

Metabolomics is more beneficial than proteomics, transcriptomics, and genomics. As the downstream products of gene and protein activity, metabolites more directly reflect physiological processes and other components of the living phenotype (Rinschen et al., 2019). Plant metabolites are grouped into primary and secondary classes. Primary metabolites are vital for growth and physiological functions, whereas secondary metabolites contribute mainly to defense responses during abiotic stress (Salam et al., 2023). Metabolomics, an advanced profiling technique that identifies all metabolites in plant cells, has become instrumental in elucidating the mechanisms underlying salinity and drought-stress responses in sugarcane. Salt-tolerant and sensitive sugarcane varieties were cultivated under salinity and drought stress to investigate secondary metabolites associated with stress adaptation. The salt-tolerant variety exhibited significant proline accumulation and reduced Na⁺ content in plant leaves (Chiconato et al., 2019). Elevated levels of phenolic acids, anthocyanins, and flavones have been shown to enhance drought and salinity resistance in sugarcane plants (Ali et al., 2019). Furthermore, during salinity stress, the physiological and developmental changes in sugarcane-developing buds are accompanied by increased hydrogen peroxide production, tissue accumulation of Cl⁻ and Na⁺, reduced K⁺ and Ca²⁺ levels, lower Ca²⁺: Na⁺ and K⁺: Na⁺ ratios, and enhanced osmolyte synthesis (Rasheed et al., 2016).

Metabolomics analysis identified reduced sucrose content and elevated reducing sugar concentrations (glucose and fructose) in sugarcane cultivars exposed to drought and salinity conditions (Vital et al., 2017). In the sugarcane variety RB867515, drought stress elicited a significant increase in glucose content, while both xylose and inositol levels rose under drought and salinity stress. Significant accumulation of several amino acids, i.e., tryptophan, phenylalanine, tyrosine, leucine, valine, proline, glutamine, lysine, isoleucine, asparagine, and glycine was observed in the RB867515 and RB855536 sugarcane cultivars.

## 6 Mitigation strategies of salinity and drought stress in sugarcane plants

Sugarcane plants experience significant yield loss due to combined stress, which leads to ionic toxicity, osmotic imbalance, and oxidative damage. Mitigation approaches have been applied to mitigate crop yield losses. Farming systems continually shape the outcomes of advances in agronomic approaches and of improvements in high-performing plant cultivars through biotechnological applications (Mariani and Ferrante, 2017). Applying salinity and drought stress-tolerant varieties with location-specific agronomic practices, early sugarcane plantation, sett priming or exogenous application of biostimulants, press mud, mulching, soil amendment with organic manure, bagasse-derived biochar and synthetic fertilizers, plant growth promoting rhizobacteria, etc., strategies for improving soil water retention, reducing salt toxicity, enhancing microbial activity, and maintaining crop productivity (**Figure 1**). The conversion of sugarcane processing residues into biochar, compost, soil amendments, and other value-added products may couple field-level salinity and drought mitigation with waste valorization, resource recycling, and the progressive development of circular sugarcane biorefinery systems (Wang et al., 2026b). This strategy balances photosynthetic responses, prevents ion toxicity, and sustains sugar yields despite adverse environmental conditions. The implementation of stress-tolerant cultivars, along with location-specific agronomic approaches, resulted in a significant improvement over traditional farmers’ practices (Sheoran et al., 2021).

Seed priming is a pre-sowing technique that involves controlled hydration and treatment of seeds to activate metabolic processes and enhance stress resilience. It is highly effective in sugarcane, where hydropriming, osmopriming, and halopriming boost germination, cellular repair, and stress-related enzyme activities to mitigate stress conditions. Exogenous applications of salicylic acid (SA), silicon (Si), or abscisic acid (ABA) effectively mitigate salt and drought stress in sugarcane plantlets. These treatments enhance antioxidant enzyme activities, boost osmolyte accumulation (proline and soluble sugars), improve K^+^/Na^+^ ratios, and protect photosynthetic pigments, thereby enabling better growth and development.

Foliar application of ABA maintains antioxidant defense systems. It assists stomatal regulation and increases the expression of stress-responsive genes to prevent water loss and cellular damage. The addition of press mud or biochar to soil/medium improves water retention and nutrient availability and reduces rhizosphere salinity. Applying organic manure alongside NPK fertilizers significantly reduces sodium absorption in leaves and promotes seed emergence. A layer of straw on the soil surface creates a protective barrier that reduces evaporation of excess water and controls the absorption of salts from shallow groundwater into the topsoil.

## 7 Conclusions

Adverse climatic factors impact cane crop production. Tolerance mechanisms involve Na⁺ exclusion, antioxidant activation, osmolyte accumulation, and stress-protein expression. While advanced breeding marker-assisted selection, transformation, CRISPR/Cas9, and omics have improved stress resilience, further gains require exploring wild relatives and landraces for novel alleles. Future sugarcane improvement may benefit from integrating genomic, transcriptomic, metabolomic, high-throughput phenotypic, and environmental data within AI-assisted genomic-enviromic prediction frameworks, thereby improving the prediction of genotype performance under dynamic salinity, drought, and combined-stress scenarios.
